# iNGNN-DTI: prediction of drug–target interaction with interpretable nested graph neural network and pretrained molecule models

**DOI:** 10.1093/bioinformatics/btae135

**Published:** 2024-03-06

**Authors:** Yan Sun, Yan Yi Li, Carson K Leung, Pingzhao Hu

**Affiliations:** Department of Biochemistry, Western University, London, ON, N6G 2V4, Canada; Department of Computer Science, University of Manitoba, Winnipeg, MB, R3T 2N2, Canada; Department of Computer Science, Western University, London, ON, N6G 2V4, Canada; Division of Biostatistics, University of Toronto, Toronto, ON, M5T 3M7, Canada; Department of Computer Science, University of Manitoba, Winnipeg, MB, R3T 2N2, Canada; Department of Biochemistry, Western University, London, ON, N6G 2V4, Canada; Department of Computer Science, University of Manitoba, Winnipeg, MB, R3T 2N2, Canada; Department of Computer Science, Western University, London, ON, N6G 2V4, Canada; Division of Biostatistics, University of Toronto, Toronto, ON, M5T 3M7, Canada; Department of Oncology, Western University, London, ON, N6G 2V4, Canada; Department of Epidemiology and Biostatistics, Western University, London, ON, N6G 2V4, Canada; The Children’s Health Research Institute, Lawson Health Research Institute, London, ON, N6A 4V2, Canada

## Abstract

**Motivation:**

Drug–target interaction (DTI) prediction aims to identify interactions between drugs and protein targets. Deep learning can automatically learn discriminative features from drug and protein target representations for DTI prediction, but challenges remain, making it an open question. Existing approaches encode drugs and targets into features using deep learning models, but they often lack explanations for underlying interactions. Moreover, limited labeled DTIs in the chemical space can hinder model generalization.

**Results:**

We propose an interpretable nested graph neural network for DTI prediction (iNGNN-DTI) using pre-trained molecule and protein models. The analysis is conducted on graph data representing drugs and targets by using a specific type of nested graph neural network, in which the target graphs are created based on 3D structures using Alphafold2. This architecture is highly expressive in capturing substructures of the graph data. We use a cross-attention module to capture interaction information between the substructures of drugs and targets. To improve feature representations, we integrate features learned by models that are pre-trained on large unlabeled small molecule and protein datasets, respectively. We evaluate our model on three benchmark datasets, and it shows a consistent improvement on all baseline models in all datasets. We also run an experiment with previously unseen drugs or targets in the test set, and our model outperforms all of the baselines. Furthermore, the iNGNN-DTI can provide more insights into the interaction by visualizing the weights learned by the cross-attention module.

**Availability and implementation:**

The source code of the algorithm is available at https://github.com/syan1992/iNGNN-DTI.

## 1 Introduction

Drugs typically refer to small molecules, while targets often refer to macromolecules such as proteins. A drug may change the function of a biological target when it binds to the target, which is known as drug–target interaction (DTI) (Sachdev and Gupta, 2019). The prediction of DTI plays a key role in drug discovery. Since it is time-consuming and expensive to identify the DTI pairs through biological assays, many computer-assisted methods have been developed (Ou-Yang *et al.,* 2012). With the success of deep learning in various areas, deep learning-based DTI prediction methods have exploded in recent years (Wen et al., 2017).

The base framework of deep learning methods for DTI involves encoding molecules and targets separately through two branches. Subsequently, the output features from these two branches are concatenated and fed into a classifier constructed using a fully connected network. Various deep learning models are utilized for feature representation learning when different raw representations for drugs and proteins are used (Bronstein *et al.* 2017, Öztürk *et al.* 2018, Huang *et al.* 2021a, 2021b, Yang et al., 2021). DeepDTA (Öztürk *et al.* 2018) uses a three-layer convolutional neural network (CNN) on the simplified molecular-input line-entry system (SMILES) string of drug molecules and the amino acid sequence of protein targets. In contrast, MolTrans (Huang *et al.* 2021a, 2021b) utilizes the transformer encoder, a module known for its excellent performance in natural language processing (NLP) tasks, on both sequence data. However, the sequence data loses the structural information of the molecule. To address this limitation, approaches that directly learn representations from the molecular graph have been developed. The graph, constructed by nodes representing atoms and edges representing bonds, offers a more natural representation of molecules. Various graph neural networks (GNNs), such as graph convolutional networks (GCN) and graph attention network (GAT), have been implemented in DTI tasks (Jiang *et al.* 2020, Nguyen *et al.* 2021). A deeper review of different deep learning approaches for compound–protein interaction prediction can be found in Jiang *et al.* (2020). However, most methods focus on encoding drugs with GNNs, with limited attempts to encode protein targets using GNNs. The challenge in representing proteins as graphs arises from the fact that the structures of most proteins are unknown. The recent breakthrough achieved by Alphafold2 (Jumper *et al.,* 2021) in predicting protein structures has eased this challenge, allowing the incorporation of graph data for proteins in DTI tasks. Our approach involves generating protein structures with Alphafold2 and applying GNNs to both drugs and targets.

Interpretability is a challenge to the DTI task. Most methods learn representations of drugs and proteins with deep learning models, but lack the interpretation of interactions between the substructures of the drugs and targets (Schenone *et al.,* 2013). ML-DTI adds a mutual learning module between the drug encoder and the target encoder (Yang *et al.* 2021). The mutual learning module calculates attention on each atom of the drug. MolTrans designs a pairwise interaction module after the encoder (Huang *et al.* 2021a, 2021b). It decomposes drugs and proteins into substructures and calculates the interaction between every two substructures. DrugBAN develops a novel bilinear interaction module to capture the interaction local structure (Bai *et al.,* 2023). The methods are all realized using the protein sequence data. However, the sequence data loses detailed local structural information, which is important to the binding site. Furthermore, the number of molecules in the DTI datasets is tiny compared to the scale of the chemical space. The problem may limit the application of the DTI model to the new drugs or targets that are unseen in the training sets.

To address the above-mentioned limitations, we propose to build an interpretable nested graph neural network for the drug–target interaction prediction (iNGNN-DTI) architecture based on the graph data of both drugs and proteins. We suggest using a nested graph neural network (NGNN) (Zhang and Li, 2021) as the fundamental feature extraction model to enhance the expression of the substructure. This improves the expression of the substructures because the representation of each node in the NGNN is the result of pooling the k-hop subgraph that surrounds it. After the encoder, we use a cross-attention free transformer (cross-AFT) (Zhai *et al.*, 2021) module to calculate the interaction between each pair of nodes from the drugs and targets. We combine the features learned by two pre-trained models, Chemformer (Irwin *et al.,* 2022) for drugs and ESM (Rives *et al.,* 2021) for proteins, with the output of the NGNN in order to further enhance the expression of the features.

The contributions of our work include: (i) the proposed method utilizes the k-subgraph GNN extractor layer from the NGNN to encode the graph data. This particular layer has demonstrated superior performance compared to a standard GNN layer; (ii) the method proposes to enhance performance by integrating the features of targets and drugs that have been extracted using models pre-trained on other large-scale unlabeled datasets; (iii) we propose an interaction module on the graph neural network for the DTI task, and the experiment results indicate that it performs better than the vanilla GNN.

## 2 Materials and methods

### 2.1 Datasets

We evaluate our method on three popularly used benchmark datasets: Davis (Davis *et al.,* 2011), KIBA (He *et al.,* 2017), and BIOSNAP (http://snap.stanford.edu/biodata) (Zitnik *et al.,* 2018). The Davis and the KIBA datasets are both for the kinase protein family. The Davis dataset indicates the activity of the drug with the equilibrium dissociation constant $K_{d}$ value. The value of the KIBA dataset is the combination of dissociation constant $K_{d}$, inhibitory constant $K_{i}$ and the half maximal inhibitory concentration ${IC}_{50}$. The two datasets need preprocessing to generate the binary label, and we follow the analysis done in previous works (He *et al.* 2017, Huang *et al.* 2021a, 2021b). For the Davis dataset, the $K_{d}$ value is transformed into log space as ${pK}_{d}$:

(1) pKd=-log10Kd1e9.

We set samples with ${pK}_{d}\geq 7$ as binding samples. For the KIBA dataset, the threshold is 12.1, and samples with a KIBA score larger than the threshold are set as positive samples. BIOSNAP collects high-throughput experiment results from diverse resources. Only positive pairs are provided by the BIOSNAP dataset, and we randomly sample unseen drug–target pairs with the same number of positive pairs as in the negative samples. The statistics of all datasets are shown in Table 1.

**Table 1.** Summary of the datasets.^a^

	No. of Drugs	No. of proteins	No. of interactions	No. of positives	No. of negatives
Davis	68	442	30 056	1506	28 550
KIBA	2068	229	118 254	22 729	95 525
BIOSNAP	4510	2180	27 428	13 817	13 611^a^

a Some of the negative pairs were removed during preprocessing the data.

### 2.2 Input data representation

Both drugs and targets can be represented in two different forms: sequence data and graph data. This section outlines the methods used to construct graph inputs for drugs and targets using their respective sequence data.

#### 2.2.1 Drug molecule representation

The sequence data of the drug molecule is the SMILES string in which each character represents atoms or bonds of the molecule. To represent drug molecules as graphs, a common approach is to convert their chemical notations into graph representations. This involves mapping atoms to nodes and chemical bonds to edges. We pass the SMILES string to RDKit (Landrum, 2016) tool to generate the drug molecule graph. We initialize the nodes of the drug molecule graph with the same feature as DGraphDTA (Jiang *et al.,* 2020). The features are listed in Table 2 and the total dimension of the atom feature is 78.

**Table 2.** Atom features in the drug molecule.

Feature name	Dim	Description
Atom element	44	One-hot encoding of atom element
Degree of the atom	11	One-hot encoding of the degree of atoms
Number of Hydrogens	11	Number of hydrogen bonds
Number of implicit Hydrogens	11	Number of implicit hydrogen bonds
Aromatic	1	The atom is aromatic or not

#### 2.2.2 Target representation

**Figure 1.**
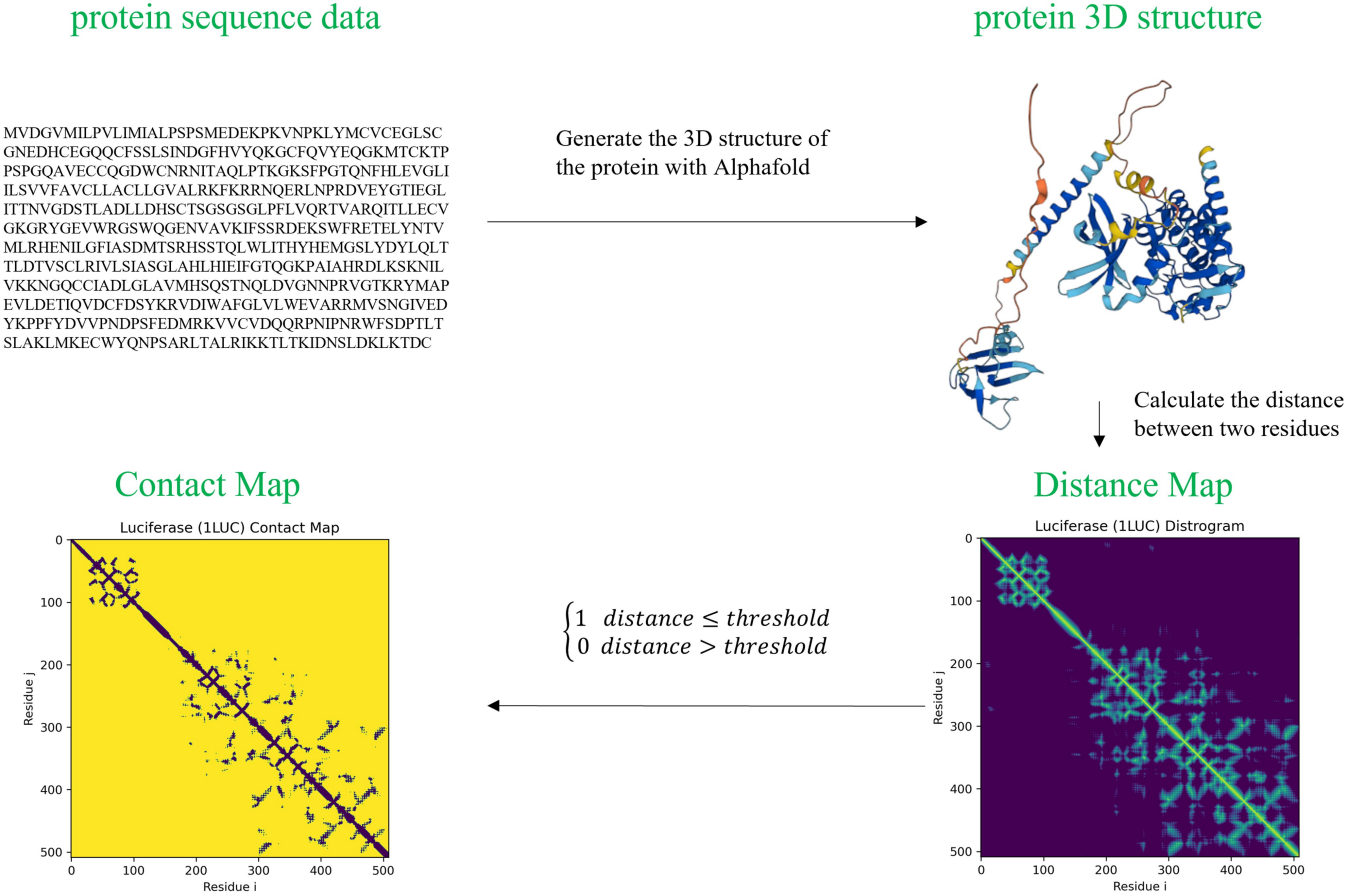
The process of the generation of protein graph with Alphafold2.

Each character in the target sequence data corresponds to an amino acid. The graph representations can be constructed by considering the structural characteristics of the amino acids and their interactions. Unlike the drug molecule, the generation of target molecule structures are complicated. The state-of-the-art method for predicting the structure of the protein is Alphafold2 (Jumper *et al.,* 2021). Alphafold2 takes the amino acid sequence as input and then outputs the three-dimensional structure of a protein. As shown in Fig. 1, we obtain the structural information of targets in the Protein Data Bank (PDB) file format from the output of Alphafold2, and then we calculate the Euclidean distance between the $C_{\alpha }$ atoms in amino acids to generate the distance map of a protein. We generate the binary contact map by setting a threshold on the distance map. In this work, we set the threshold as 8 Å based on the suggestion made by Duarte *et al.* (2010). Other two alternative thresholds are also considered (see Supplementary Table S1). The node feature of the protein graph is the same as Jiang *et al.* (2020). The specific description of the feature is shown in Table 3.

**Table 3.** Protein molecular graph feature initialization.

Feature name	Dim	Description
Residual symbol	21	One-hot encoding of the residue
PPM	21	Position Probability Matrix
Aliphatic	1	The residue is aliphatic or not
Aromatic	1	The residue is aromatic or not
Polar neutral	1	The residue is polar neutral or not
Acidic charged	1	The residue is acidic charged or not
Basic charged	1	The residue is basic charged or not
Residue weight	1	The weight of the residue
Dissociation constant for the –COOH group	1	The negative of the logarithm of the dissociation constant for the –COOH group
Dissociation constant for the –NH3 group	1	The negative of the logarithm of the dissociation constant for the –NH3 group
Dissociation constant for any other group in the molecule	1	The negative of the logarithm of the dissociation constant for any other group in the molecule
The pH at the isoelectric point	1	The pH at the isoelectric point
Hydrophobicity of residue (pH = 2)	1	Hydrophobicity of residue (pH = 2)
Hydrophobicity of residue (pH = 7)	1	Hydrophobicity of residue (pH = 7)

Position Probability Matrix (PPM) is a simplified feature of the Position-Specific Scoring Matrix (PSSM), which is widely used to evaluate the similarity between protein sequences (Cheol Jeong *et al.,* 2010). The PPM is generated based on the result of multiple sequence alignment (MSA). MSA aligns a query protein with thousands of protein sequences and outputs an aligned sequence array (Edgar and Batzoglou, 2006). The PPM calculates the occurrence probability of each residue on each point. The calculation of the PPM can be represented as:

(2) Mr,k=Fr,k+p4N+p,

where $F\in N^{R\times K}$ is the frequency matrix, R is the number of residues, and K is the length of the protein sequence. Each row corresponds to a type of residue. $F_{r,k}$ represents the occurrence frequency of the residue r at the position k. p is a pseudocount. N is the total number of aligned sequences.

### 2.3 Methods

**Figure 2.**
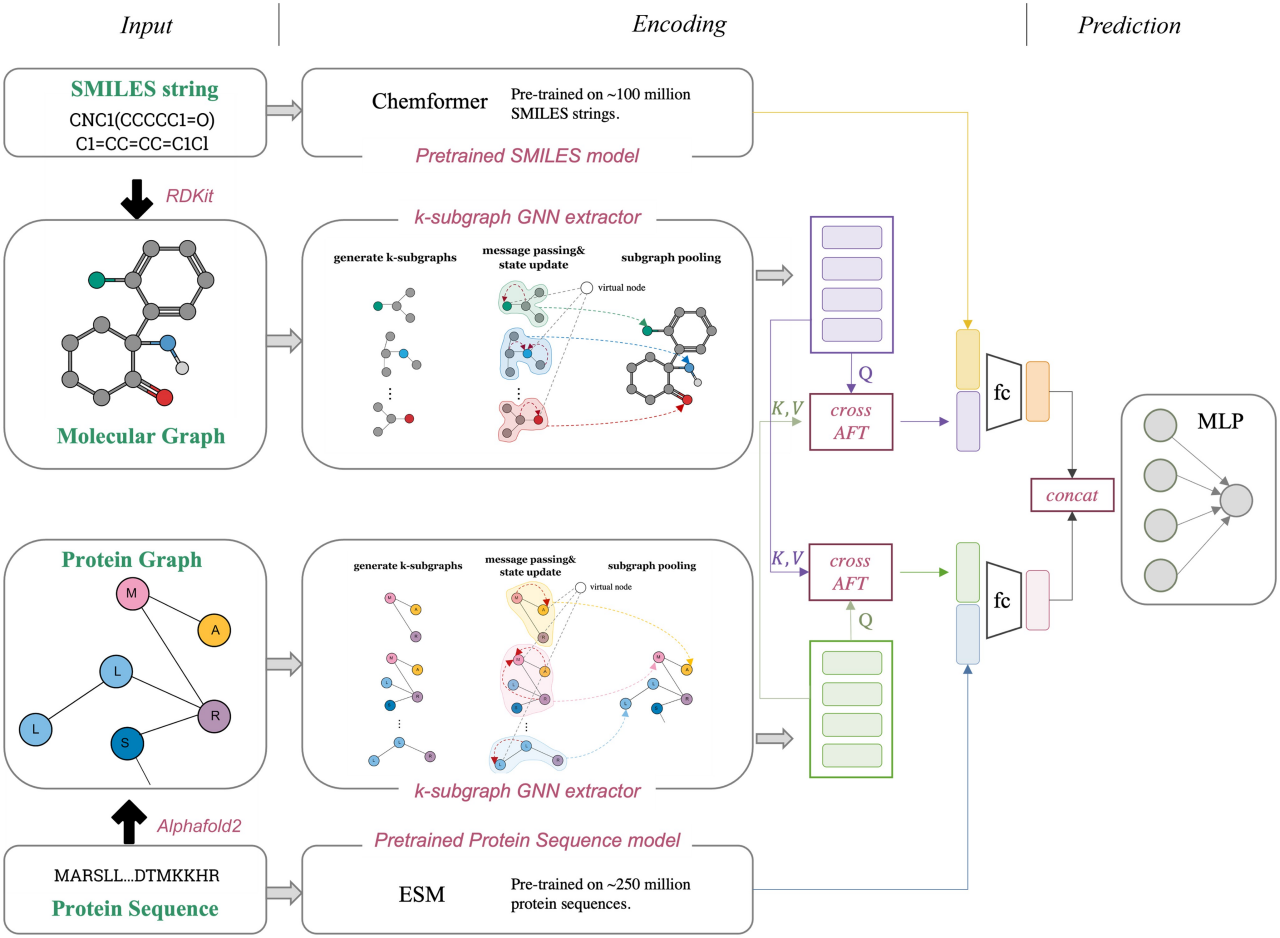
Model architecture of iNGNN-DTI. The SMILES string and the protein amino acid sequence are transformed into the graph data in the Input section. The Encoding section uses the k-subgraph GNN extractor to encode the graph data while using the Chemformer and ESM models to encode the SMILES string and amino acid sequence, respectively. To learn the interaction information, the cross-AFT module connects to the GNN extractor. The final representation of the drug and the protein is created by concatenating the output of the cross-AFT module with the global feature created by the unsupervised model. In the end, the two representations are concatenated and passed to an MLP module for the prediction of the interaction.

Figure 2 illustrates the proposed model architecture, which can be divided into three main components: Input, Encoding, and Prediction. In the Input part, the graph data is generated along with the input sequence data. The Encoding part comprises three models. The Chemformer (Irwin *et al.,* 2022) and ESM (Rives *et al.,* 2021) models are pre-trained models used for extracting features from the sequence data of drugs and targets, respectively. Additionally, the GNN part utilizes a k-subgraph GNN extractor, as proposed in NGNN (Zhang and Li 2021), which begins by generating k-hop subgraphs. Therefore, each node is a representation of a subgraph. The method creates the graph representation in a hierarchical fashion, beginning with the generation of subgraph-level representations and then constructing the representation for the entire graph. To capture the interaction information between drugs and targets, a cross-attention free transformer (cross-AFT) module is connected to the GNN model. Finally, in the Prediction part, all the extracted features are integrated and fed into a multilayer perceptron (MLP) module for making predictions. The new algorithm is named iNGNN-DTI. Further details about each module will be presented in the next subsections.

#### 2.3.1 k-subgraph GNN extractor layer with virtual node

To encode the graph data, we utilize the k-subgraph GNN extractor, as introduced in NGNN ( Zhang and Li, 2021). Traditional GNN models like GCN have limitations in expressing substructures when the graph is nested rather than a tree structure (Chen *et al.,* 2022). The k-subgraph GNN extractor addresses this limitation by updating the representation of each node using the k-hop subgraph surrounding that node, resulting in more powerful representations compared to base GNNs. The implementation of the k-subgraph GNN extractor involves incorporating a subgraph pooling layer on top of the base GNNs. This pooling operation aggregates information from the k-hop subgraph and propagates it to update the node representations.

Here, we denote the graph data as G=(V,E), where V is the set of nodes and E is the set of edges. An intermediate node representation will be generated first with a base layer based on GCN. The intermediate representation of the node u at the layer l+1 is calculated as:

(3) hul+1=σ∑j∈NuWlhjl, 

where $h_{u}^{\left(l+1\right)}\in R^{d}$ denotes the representation of the node u and d is the size of the dimension. σ(·) is a nonlinear activation function. N(u) is the set for the neighborhoods of the node u. $W^{l}\in R^{d\times d}$ is the weight of the layer l. The final representation is obtained through performing a subgraph pooling on each node following the base GNN layers. The pooling operation is calculated as:

(4) hu=∑k∈Suhk,

where S(u) is the set of nodes in the k-subgraph of node u.

A virtual node is an additional node inserted into the graph. Virtual edges are created to link the virtual node to all other nodes in the graph (Gilmer *et al.,* 2017). After the update of the node representation, the virtual node collects the information of all nodes and scatters it back to each node before the calculation of the next layer. The virtual node operation is calculated as:

(5) vl=σvl-1W0l+∑i∈VhilW1l, 

(6) hul+1=hul+1+vl, 

where $v^{\left(l\right)}$ represents the virtual node representation at layer l, and $W_{0}^{\left(l\right)},\;W_{1}^{\left(l\right)}\in R^{d\times d}$ are two learnable weight matrices.

#### 2.3.2 Interaction module

The interaction module is designed to capture the molecular interactions between drugs and their target proteins, highlighting key atoms in the drug molecule and showcasing the attention patterns in the target during the interaction. The interaction module is implemented with an attention-free transformer (AFT) ( Zhai *et al.*, 2021). Different from classical transformers (Vaswani *et al.* 2017), AFT proposes a transformer operation without scale dot product attention. Given an input X, AFT operates as:

(7) Yt=σqQt⊙∑t'=1Texp⁡Kt'+wt, t'⊙Vt'∑t'=1Texp⁡Kt'+wt, t', 

where Q, K, and V are query, key, and value matrices generated by linear transformations: $Q=XW^{Q}$, $K=XW^{K}$ and $V=XW^{V}$. $w\in R^{T\times T}$ is the pair-wise position biases learned during training. t indicates the position. $\sigma_{q}$ is the sigmoid function. The operation is revised to a cross-AFT in this work.

We denote the outputs of the GNN model as $H_{\mathrm{drug}}\in R^{N_{\mathrm{drug}}\times d}$ and $H_{\mathrm{prot}}\in R^{N_{\mathrm{prot}}\times d}$ for the drug and target, respectively. $N_{\mathrm{drug}}$ is the number of nodes in the drug and $N_{\mathrm{prot}}$ is the number of nodes in the protein. The cross-AFT on the drug branch can be calculated as:

(8) Qdrug, Kprot, Vprot=HdrugWdrugQ, HprotWprotK, HprotWprotV, 

(9) Ydrugn=σqQdrugn⊙∑n'=1Nprotexp⁡Kprotn'+wn,n'⊙Vprotn'∑n'=1Nprotexp⁡Kprotn'+wn,n',

(10) Ydrug=Ydrug+Hdrug,

where ⊙ is the element-wise product. $w\in R^{N_{\mathrm{drug}}\times N_{\mathrm{prot}}}$ represents the pair-wise bias which is learned during the training phase. The cross-AFT process on the target branch involves similar steps, where the query matrix is generated using $H_{\mathrm{prot}}$, and the key and value matrices are derived from $H_{\mathrm{drug}}$.

#### 2.3.3 Merge features extracted from the pre-trained models

Molecule features extracted by pre-trained models may include fundamental properties information. We apply Chemformer (Irwin *et al.,* 2022) for feature extraction of drug molecules and ESM (Rives *et al.,* 2021) for proteins. For both of them, the input is the sequence data. Chemformer is a BART (Lewis *et al.*, 2020) model which is pre-trained on >100 million SMILES strings. The length of the output feature is 512. ESM is a 34-layer transformer model, and it is trained with 250 million amino acid sequences. The length of the output feature is 768. Both features are mapped to 128D with the fully connected (FC) layer.

We denote the feature of the SMILES string as $P_{\mathrm{drug}}$ and the feature of the target as $P_{\mathrm{prot}}$. The two features are fused with the outputs of the interaction module with the fully connected (FC) layer to generate the final representations:

(11) Fdrug=FCYdrug·Pdrug, 

(12) Fprot=FCYprot·Pprot,

where [·] denotes the concatenation operation. The two final representations are finally joined together and given to an MLP module to predict the interaction:

(13) output=MLPFdrug·Fprot

### 2.4 Baseline models and model evaluation

For the evaluation of the proposed model performance, four baseline models are used for comparison. DeepDTA (Öztürk *et al.* 2018), ML-DTI ( Yang *et al.*, 2021) and Moltrans (Huang *et al.* 2021a, 2021b) are three methods using sequence data as inputs. DeepDTA uses two 3-layer CNN models for both drugs and targets. Moltrans is a transformer model with an interaction matrix after feature extraction. ML-DTI designs an interaction module between the CNN layers. However, it only calculates the attention on the drug molecule. DGraphDTA applies GNN to extract features on the drug molecule and the target protein (Jiang *et al.* 2020). The original DGraphDTA uses PconsC4 (Michel *et al.* 2019) to construct the protein structure, whereas our test will use the Alphafold2 result. All methods are tested with the suggested hyperparameters from the original studies.

Four widely used performance metrics for the DTI task are used in this study: area under the receiver operating characteristic (AUROC), area under precision-recall curve (AUPRC), sensitivity and specificity. Each dataset is split into train, validation, and test sets in the ratio 7:1:2. During evaluation, we conduct five independent runs on each dataset and report the mean value and standard deviation of the results.

## 3 Results

### 3.1 Performance of the DTI prediction

As shown in Table 4, our method achieves the highest performance according to AUROC and AUPRC among all methods on all three datasets. For the Davis dataset, we get improvements in AUROC of 2.3% (from 0.910 to 0.931) and AUPRC of 23.8% (from 0.382 to 0.473). For the BIOSNAP dataset, the improvements are 2.3% (from 0.913 to 0.934) and 2.4% (from 0.917 to 0.939) for AUROC and AUPRC, respectively. The improvements on the KIBA dataset are 0.3% (from 0.912 to 0.915) for AUROC and 1.3% (from 0.743 to 0.753) for AUPRC.

**Table 4.** Model performance comparison.

Method	AUROC	AUPRC	Sensitivity	Specificity
DAVIS dataset				
DeepDTA	0.892±0.0066	0.378±0.0231	0.854±0.0066	0.792±0.0291
Moltrans	0.898±0.0050	0.371±0.0067	0.865±0.0050	0.783±0.0387
ML-DTI	0.910±0.0034	0.381±0.0247	0.895±0.0034	0.795±0.0183
DGraphDTA (Alphafold2)	0.885±0.0099	0.316±0.0447	0.894±0.0099	0.724±0.0467
**iNGNN-DTI**	**0.931 ± 0.0027**	**0.473 ± 0.0167**	**0.922 ± 0.0155**	**0.802 ± 0.0240**
KIBA dataset				
DeepDTA	0.912±0.0037	0.743±0.0127	0.881±0.0056	0.780±0.0127
Moltrans	0.899±0.0022	0.691±0.0142	0.872±0.0116	0.760±0.0160
ML-DTI	0.909±0.0020	0.727±0.0108	0.878±0.0111	0.779±0.0113
DGraphDTA (Alphafold2)	0.911±0.0004	0.739±0.0043	0.881±0.0183	**0.784 ± 0.0277**
**iNGNN-DTI**	**0.915 ± 0.0016**	**0.753 ± 0.0071**	**0.888 ± 0.0107**	0.779±0.0146
BIOSNAP dataset				
DeepDTA	0.897±0.0027	0.900±0.0046	0.859±0.0089	0.786±0.0197
Moltrans	0.887±0.0034	0.881±0.0085	0.824±0.0106	0.809±0.0104
ML-DTI	0.911±0.0053	0.911±0.0112	0.851±0.0054	0.828±0.0215
DGraphDTA (Alphafold2)	0.913±0.0022	0.917±0.0024	0.858±0.0175	0.831±0.0151
** iNGNN-DTI**	**0.934 ± 0.0021**	**0.939 ± 0.0022**	**0.872 ± 0.0189**	**0.854 ± 0.0200**

The bolded numbers represent the best results.

### 3.2 Performance on the test set with the unseen drugs and targets

In practice, it is common for new drug or targets to be discovered while their interactions have not yet been determined. To ensure DTI predictions in such cases, unseen input data is extracted from the DAVIS dataset. We process the dataset following the setting in MolTrans (Huang *et al.* 2021a, 2021b). During the split of the dataset, we randomly select 20% drugs or targets and set all related DTI samples as the test set. The remaining samples are divided into training and validation sets in a 7:1 ratio. All baselines are tested on the unseen datasets.

The results are shown in Table 5. When the test datasets do not contain any unseen samples, we observe that ML-DTI exhibits the highest performance compared to the other three baseline methods on the DAVIS dataset (Table 4). However, when considering the scenario of unseen drugs, ML-DTI demonstrates performance comparable to DeepDTA and MolTrans. Furthermore, our proposed method exhibits greater consistency in its performance. Specifically, we achieve improvements in AUROC of 2.5% (from 0.744 to 0.763) and AUPRC of 32.5% (from 0.169 to 0.224) for the unseen drug case. Similarly, for the unseen protein case, we achieve improvements in AUROC of 3.1% (from 0.840 to 0.867) and AUPRC of 14.3% (from 0.259 to 0.296).

**Table 5.** Model performance on the test set with unseen drugs and proteins.

Method	unseen drugs		unseen proteins	
	AUROC	AUPRC	AUROC	AUPRC
DeepDTA	0.736±0.0550	0.145±0.0300	0.770±0.0594	0.145±0.0300
Moltrans	0.744±0.0540	0.144±0.0370	0.778±0.0538	0.231±0.0534
ML-DTI	0.737±0.0700	0.169±0.0730	0.840±0.0357	0.259±0.0519
DGraphDTA	0.718±0.0045	0.169±0.0049	0.780±0.0478	0.164±0.0391
**iNGNN-DTI**	**0.763 ± 0.0490**	**0.224 ± 0.0640**	**0.867 ± 0.0357**	**0.296 ± 0.0534**

The bolded numbers represent the best results.

### 3.3 Interpretability analysis

To enhance the interpretability of the prediction results, we visualize the information learned by the interaction module, specifically the AFT module. Unlike explicit attention matrices, the AFT module does not directly learn an attention matrix. However, it can be interpreted as having an implicit attention mechanism through its operations. The interpretation of the AFT module with implicit attention can be understood as follows:

(14) Ydrugn,i=<adrugn,i, Vproti>,

(15) s.t. adrugn, i=σqQdrugn,iexp⁡Kprotn,i+wn∑n'=1Nprotexp⁡Kprotn',i+wn,n', i=1,2, …, d, n=1,2,…,Nprot. 

Here i is the index of feature dimension and $a_{\mathrm{drug}}^{n,\;i}\in R^{N_{\mathrm{prot}}}$ denotes the attention vector for each dimension. Two attention matrices $A_{\mathrm{drug}}\in R^{N_{\mathrm{drug}}\times N_{\mathrm{prot}}\times d}$ and $A_{\mathrm{prot}}\in R^{N_{\mathrm{prot}}\times N_{\mathrm{drug}}\times d}$ are generated and these matrices are derived by considering drugs and targets as queries, respectively. The following subsections analyze the learned attention matrices through visualization and virtual docking.

#### 3.3.1 Visualization analysis of the targets

**Figure 3.**
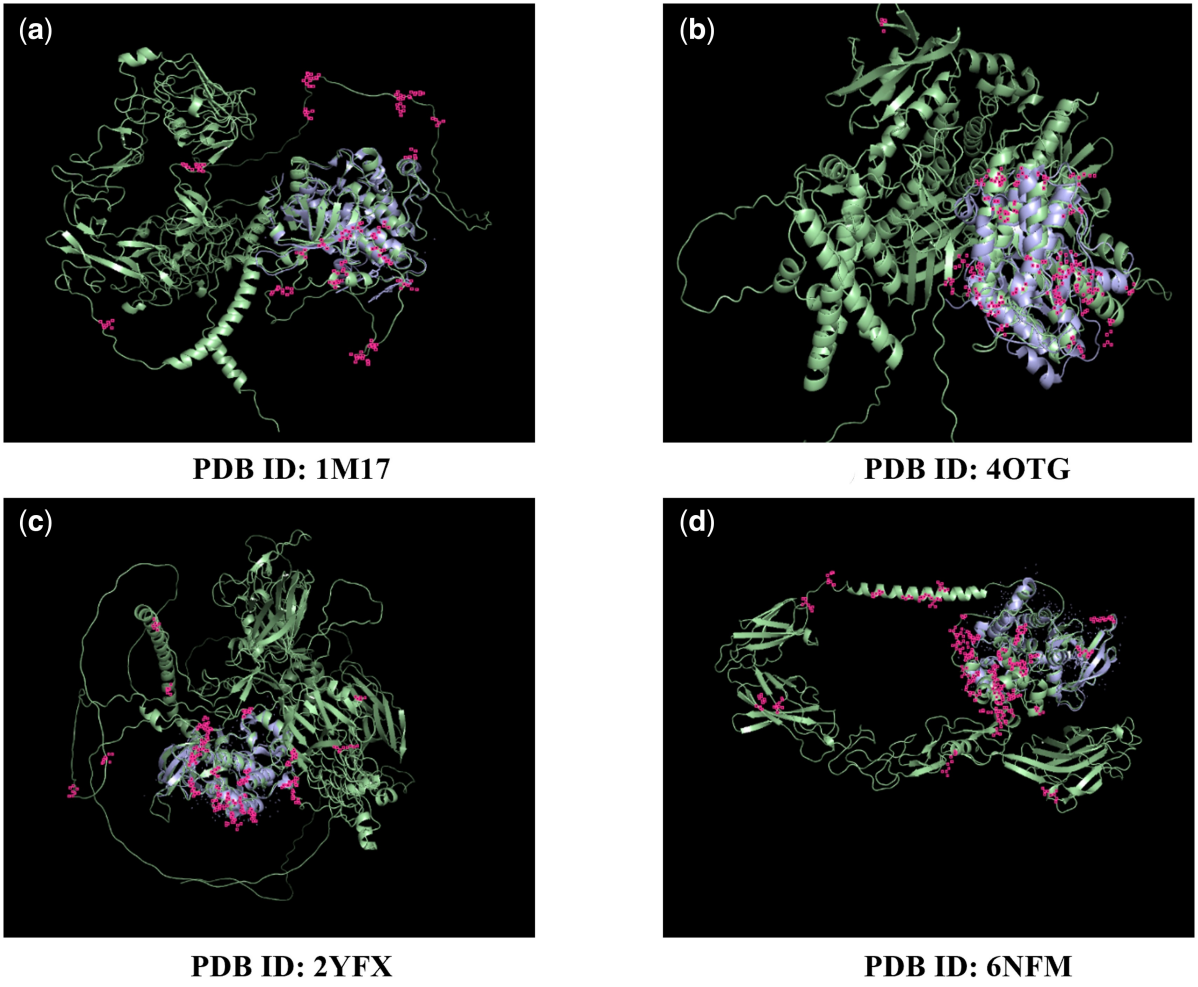
Visualization of the residues with the highest weight in each dimension on the target structure. The complete structure of the protein predicted by AlphaFold2 is denoted by the green structures, while the purple structures represent the structure sourced from the Protein Data Bank (PDB). Residues with the highest scores in each dimension are marked by pink dots.

We randomly select 4 protein targets that have both the true and predicted protein structures. We visualize the residues with the highest weight scores in each dimension, indicated by the pink dots in Fig. 3. As illustrated in Fig. 3, the green structures represent the complete protein structure generated from AlphaFold2 and are used as input into our model, whereas the purple sections indicate the reference structure obtained from the Protein Data Bank (PDB). The entire protein is notably longer than the complex segment, presenting difficulties in accurately identifying the binding site, particularly in our configuration, which lacks explicit binding site information. Among the labelled residues, we note that some residues are located in the loop regions, possibly owing to the frequent presence of such structures in proteins, and these regions show minimal differences. However, a distinct clustering of residues around the interaction regions is clearly observable. This pattern is especially prominent in the case of the 4OTG (Fig. 3b), where the majority of residues are densely located near the complex.

#### 3.3.2 Virtual docking analysis

**Figure 4.**
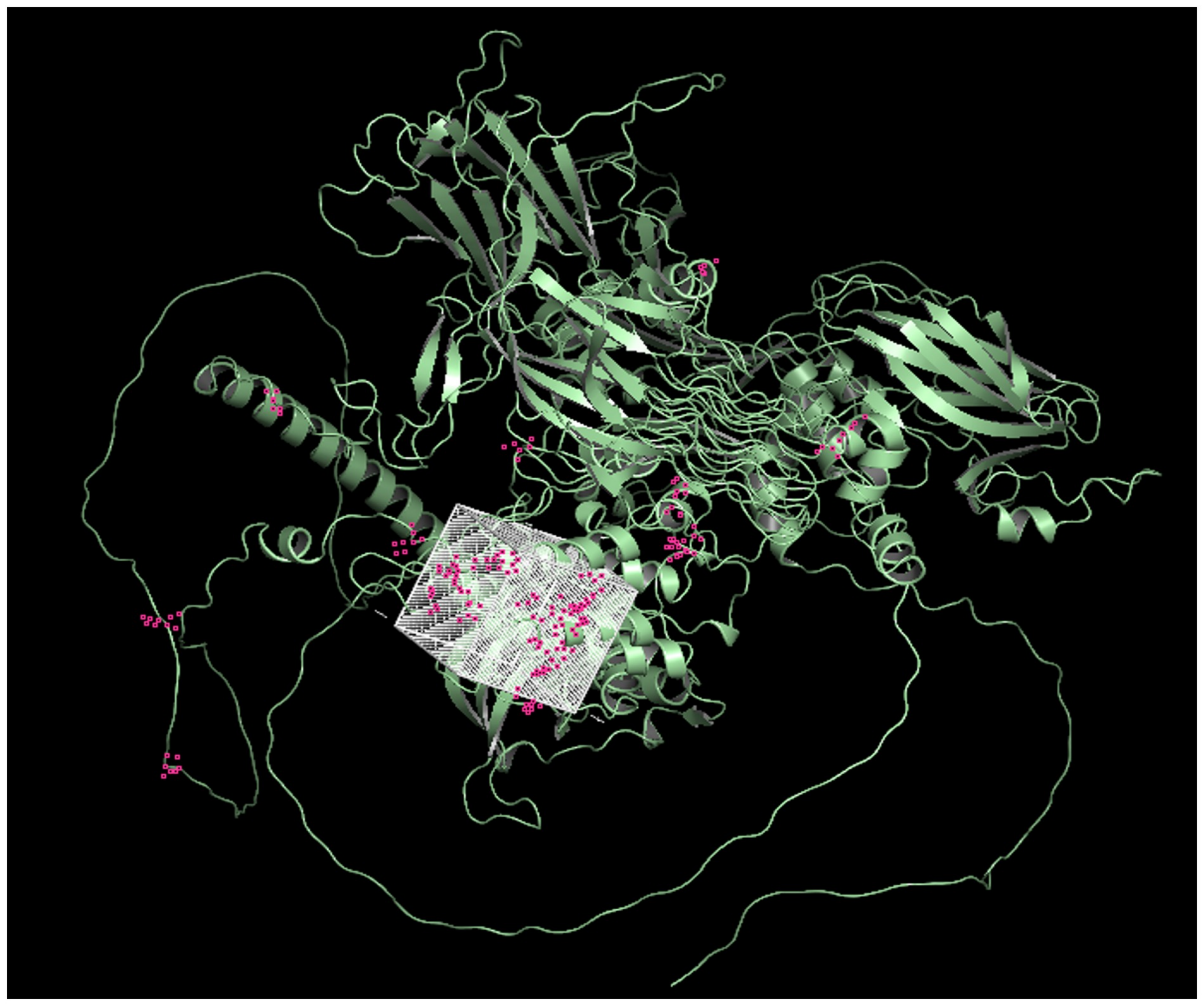
Illustration of the grid box configuration used for virtual docking on 2YFX.

**Figure 5.**
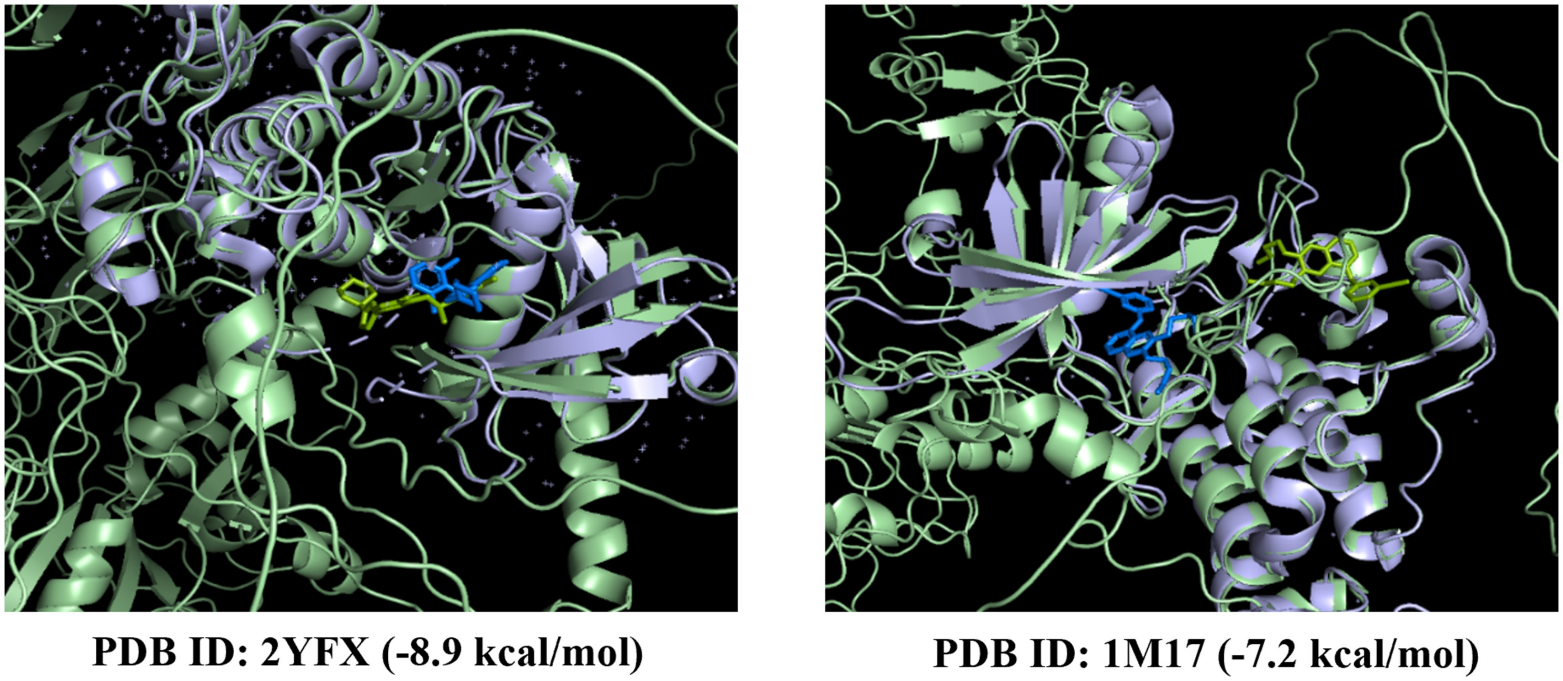
Visualization of the virtual docking result. The ligand in blue is sourced from the PDB, while the green ligand is derived from our virtual docking result.

We further perform virtual docking tests by placing the grid box across the area where most residues with higher scores are clustered. Figure 4 displays the grid box utilized for the structure 2YFX case. The virtual docking results based on Autodock Vina (Eberhardt *et al.* 2021) are shown in Fig. 5. The ligand in blue (the true target structure in purple) is sourced from the PDB, while the green ligand is derived from our virtual docking result. Both cases achieved moderate virtual docking scores (−8.9 and −7.2 kcal/mol), and for the 2YFX case, our virtual docking position closely aligns with the position provided by the PDB.

#### 3.3.3 Visualization analysis on the drug molecules

**Figure 6.**
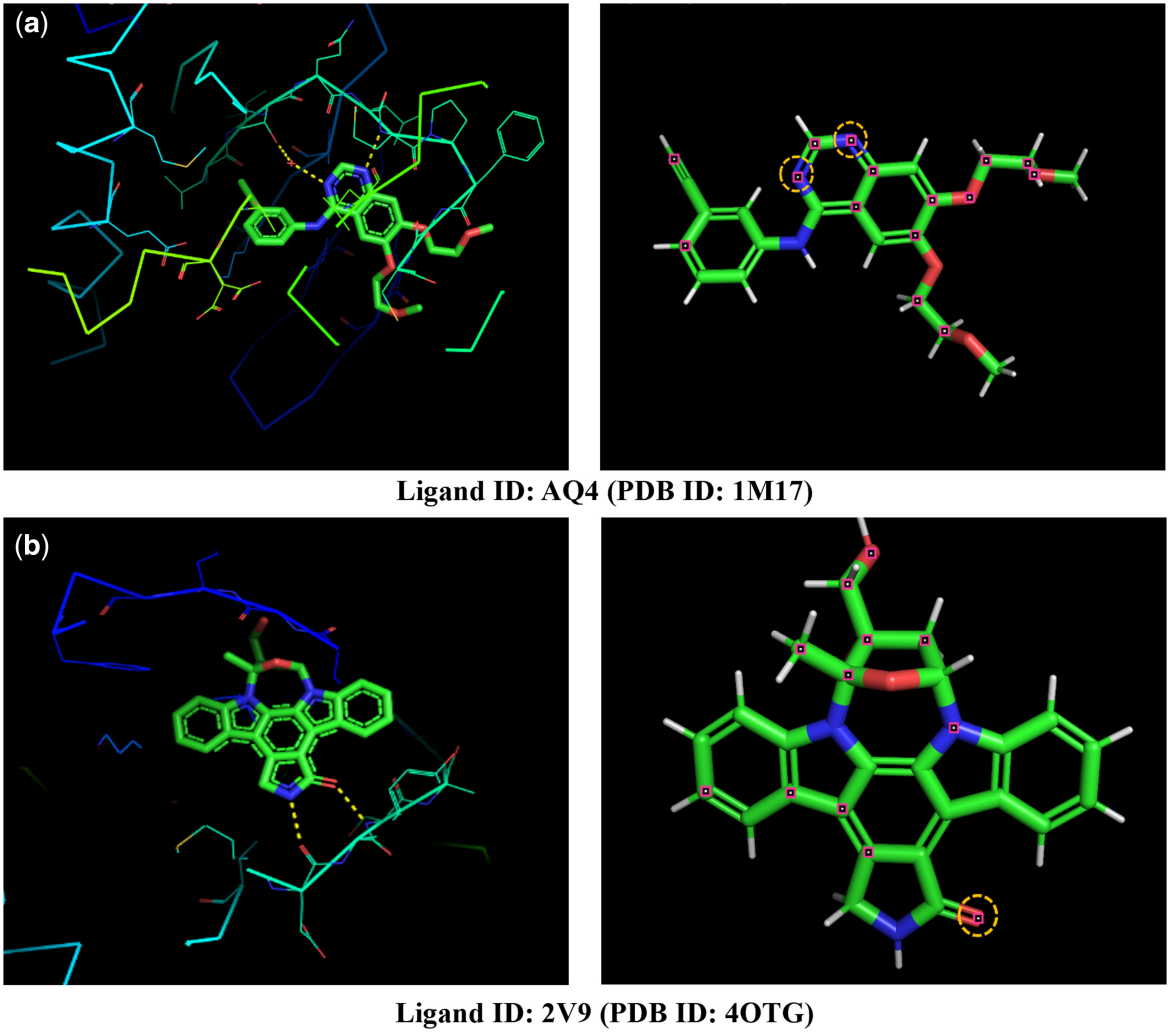
Visualization of the atoms with the highest weight in each dimension on the drug molecule. In each panel, known polar contacts are illustrated with yellow dashed lines on the left side figure. On the right side, atoms with high scores are denoted by pink points, while atoms forming bonds with the target are enclosed within yellow circles.

We visualize atoms with the highest weight on the drug molecule (Fig. 6). In the 1M17 complex, the target protein forms hydrogen bonds with N2 and N3 in the drug molecule, both highlighted by our attention mechanism. Similarly, in 4OTG, O2 and N3 engage in hydrogen bonds with the target protein, with one of them emphasized by our attention mechanism.

### 3.4 Ablation study

There are two modifications on top of the basic GNN networks: the interaction module using the cross AFT and the use of the features extracted by the unsupervised model. We conduct the ablation study to learn the effectiveness of each module using the Davis dataset.

As shown in Table 6, we observe that the cross-AFT module enhances the AUPRC result, and the incorporation of features learned by the unsupervised models proves beneficial in improving the AUROC. Combining the two modules resulted in an improvement compared to the results obtained from the vanilla NGNN. The AUROC increased by 1.3% (from 0.919 to 0.931), while the AUPRC increased by 1.9% (from 0.464 to 0.473).

**Table 6.** Ablation test on the DAVIS dataset.

No. of tests	GNN	NGNN	cross AFT	unsupervised model	AUROC	AUPRC
1	√				0.916±0.0046	0.447±0.0181
2		√			0.919±0.0037	0.464±0.0357
3		√	√		0.920±0.0051	**0.478 ± 0.0222**
4		√		√	0.927±0.0052	0.431±0.0103
5		√	√	√	**0.931 ± 0.0027**	0.473±0.0167

The bolded numbers represent the best results.

### 3.5 Coronavirus disease 2019 case study

Coronavirus disease 2019 (COVID-19) is caused by Severe Acute Respiratory Syndrome (SARS-CoV-2) (Mohanty *et al.* 2020). Numerous proteins have been discovered that have an effect on the COVID-19 virus. Our objective in this analysis is to explore the potential of repurposing drugs that interact with angiotensin-converting enzyme 2 (ACE2), a receptor protein found on the surface of cells (Kumar 2020). This protein serves as the receptor for SARS-CoV-2 to enter and infect human cells.

**Figure 7.**
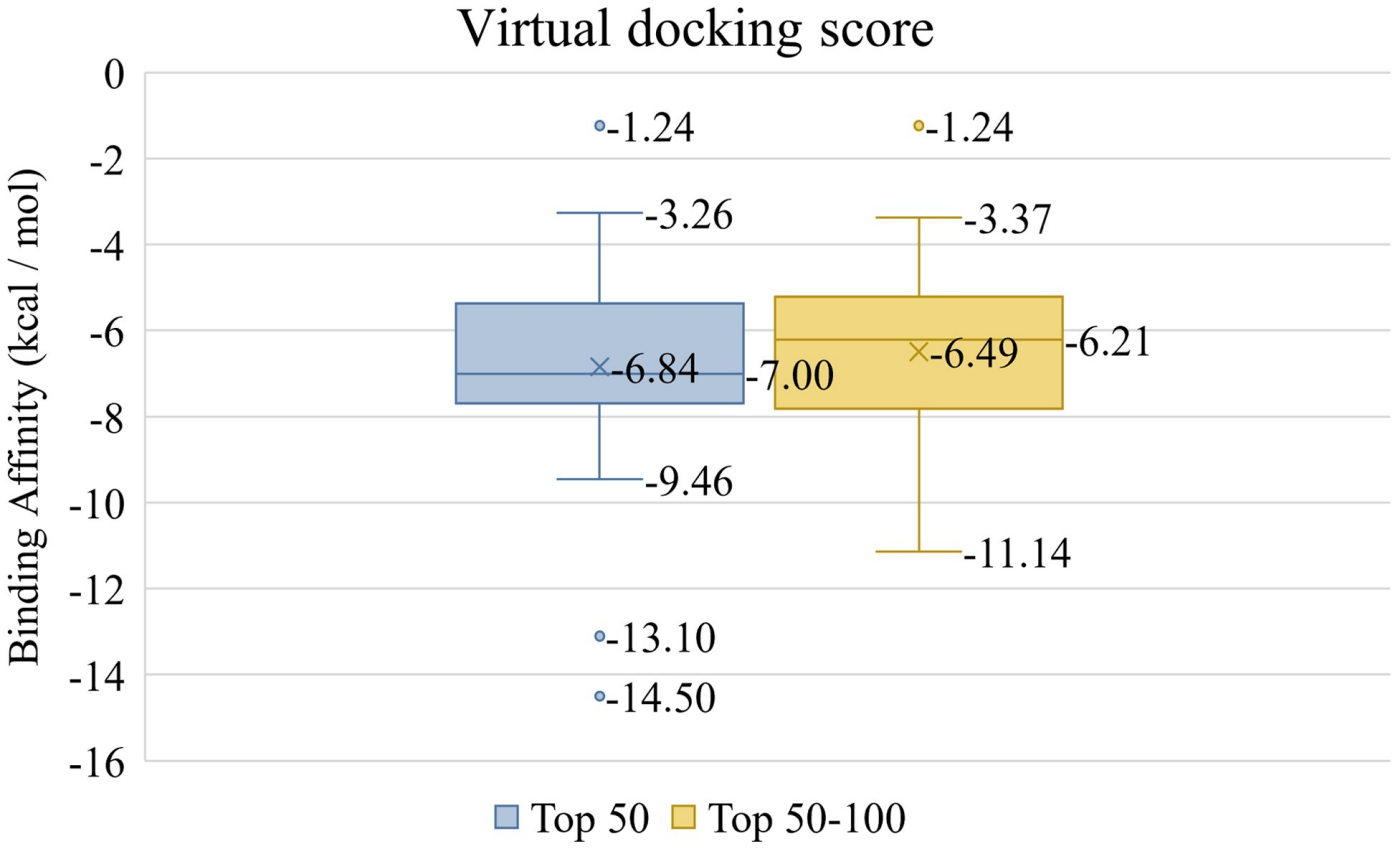
Virtual docking scores for top 50 drugs, and top 50–100 drugs.

We pre-train a model using the BIOSNAP dataset and predict the interaction between ACE2 and all 4510 drugs in the BIOSNAP dataset. Due to the limited validations of most drugs against the ACE2 target, our approach involves conducting blind virtual docking to discover potential binding sites for each drug from the whole protein structure for performance evaluation utilizing the DockConv2 (Chen *et al.* 2021). Additionally, we also performed virtual docking analysis based on the grid box across the area where most residues with higher scores are clustered following the methodology outlined above. Figure 7 illustrates the blind virtual docking scores for the generated top 50 molecules and top 50–100 molecules. The mean (median) virtual docking score obtained from DockConv2 for the top 50 drugs is −6.84 (−7.00) kcal/mol, while for the top 50–100 drugs, the score is −6.49 (−6.21) kcal/mol. The top 50 drugs showed better performance in terms of virtual docking scores.

Among the top five drugs generated by our methods, Pseudoephedrine and Ephedrine are found as inhibitors that disrupt the interactions between ACE2 and the SARS-CoV-2 receptor-binding domain of spike protein (Yu *et al.* 2021, Mei *et al.* 2021). In Yamamoto *et al.* (2022), a cell surface entry path of SARS-CoV-2 is identified which is sensitive to various metalloproteinase inhibitors. Marimastat, a metalloproteinase inhibitor, is considered a promising candidate for inhibiting COVID-19 (Yamamoto *et al.* (2022)). Furthermore, erythromycin, exhibiting the lowest binding affinity with a score of −14.5 kcal/mol, has been the subject of other studies where it is recognized as a high-potential drug for COVID-19 (Wang *et al.* 2022).

**Figure 8.**
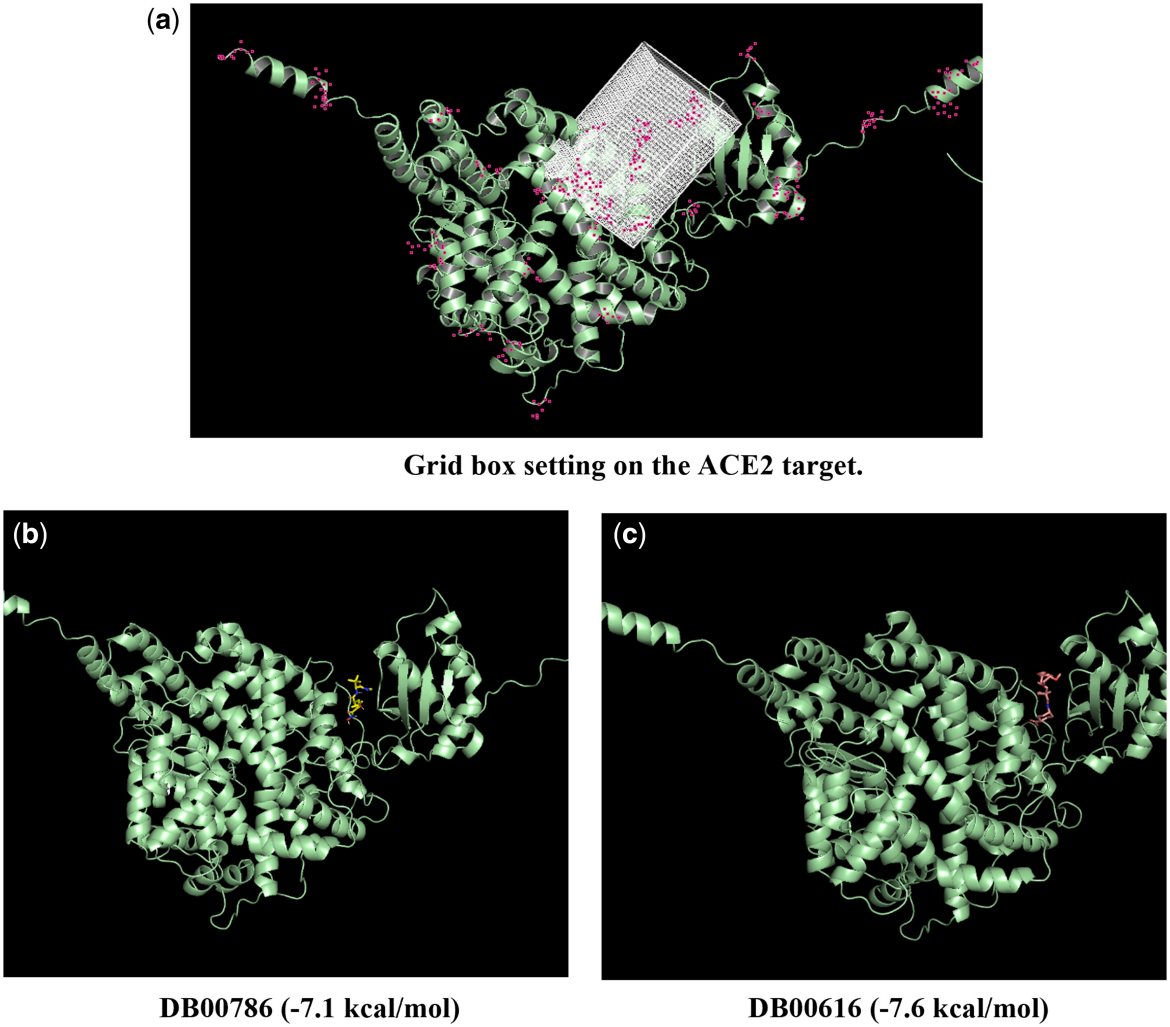
Visualization of grid box-based virtual docking results for drugs DB00786 and DB00616 on the ACE2 target.

**Figure 9.**
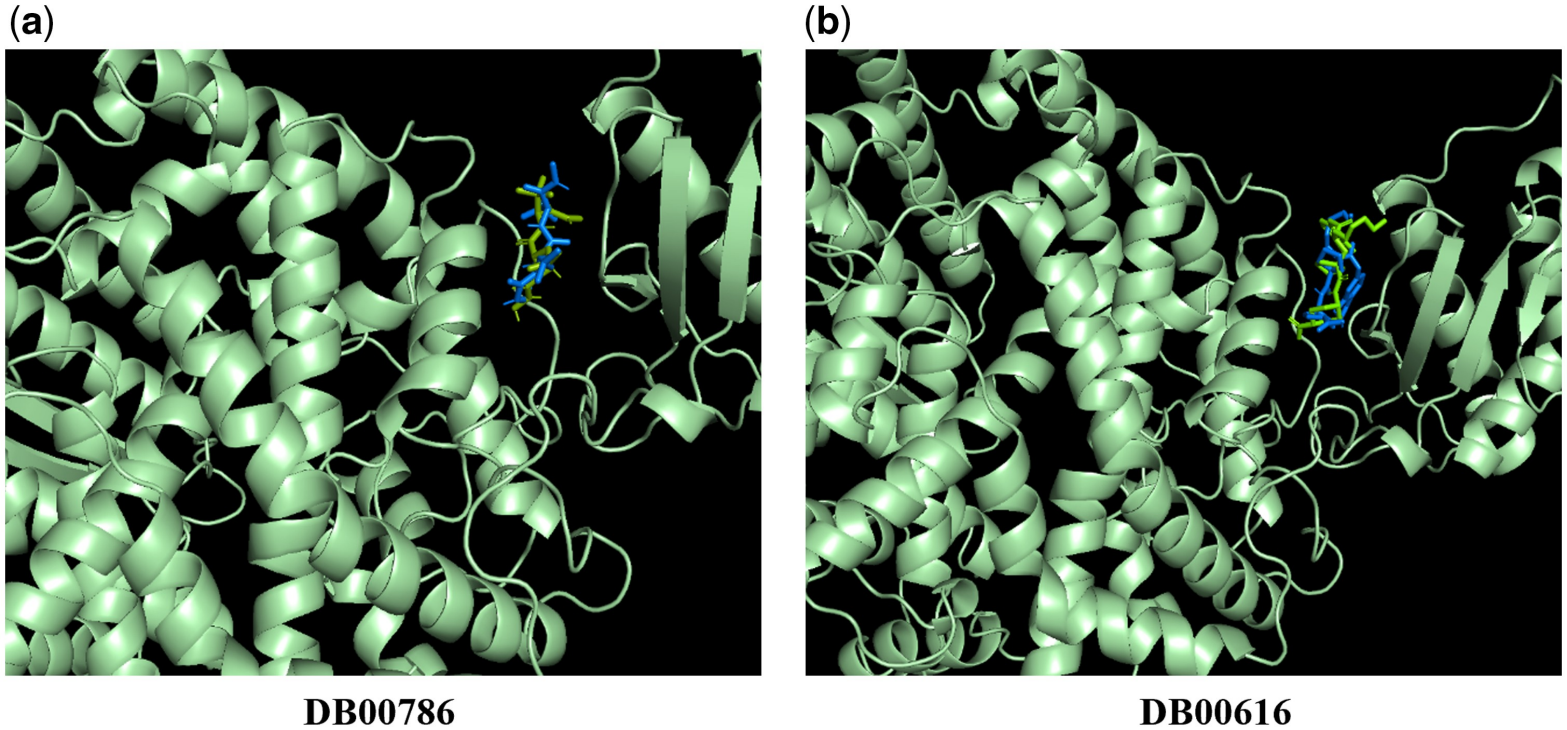
Comparison of blind docking results and our grid box-based virtual docking results. The blue ligand represents the outcome from blind docking, while the green ligand represents the results from our grid box-based virtual docking analysis.

Figure 8a illustrates the setting of the grid box on the ACE2 target. We run the virtual docking for two drugs (DB00786 and DB00616) from the top 5 drug molecules in our results. The virtual docking results are displayed in Fig. 8b and c. The virtual docking scores generated by our grid box are −7.1 kcal/mol and −7.6 kcal/mol. Additionally, we observed that our virtual docking positions closely align with the results produced by blind docking using DockCov2 (Fig. 9).

## 4 Conclusion

Our work introduces a novel interpretable nested graph neural network for the prediction of DTIs. We utilize AlphaFold2 to generate the molecular structures of the target proteins and create graph based on the structures. The model combines features extracted from 1D sequence data using pre-trained models and features obtained from the 2D molecular graph learned by the GNN. To capture the interaction information between drugs and targets, we incorporate an attention-free transformer module.

Our proposed architecture achieves superior performance compared to four baseline models across all test datasets. In a case study focused on COVID-19, we repurpose five drugs, and three of them have been identified as promising candidates for COVID-19 treatment based on previous studies. This showcases the potential of our approach in aiding drug discovery tasks.

From the interpretability analysis of our proposed model, we observe that the model can highlight certain atoms within some drugs that participate in hydrogen bonds or water-mediated bonds. However, we note that the attention weights assigned to the protein target do not always exhibit a strong correlation with the actual positions of the residues, which indicates the need for further improvement in our future work.

Future study can be performed to improve our algorithm. First, deeper analyses can be made to examine the performance of our algorithm to use both membrane and nonmembrane proteins when AlphaFold2 is used to predict their structures, although our preliminary results show that AlphaFold2 can also accurately predict the structures of most of the membrane proteins in our datasets (Supplementary Figs S1 and S2); Second, using AlphaFold2 to build the contact map is computationally extensive, it will be interesting to explore other approaches to construct the contact map from protein sequence (Supplementary Fig. S3).

## Supplementary Material

btae135_Supplementary_Data
