# Supplementary material for: iNGNN-DTI: prediction of drug–target interaction with interpretable nested graph neural network and pretrained molecule models
Source: Bioinformatics. 2024 Mar 6;40(3):btae135. doi: 10.1093/bioinformatics/btae135 (PMC10957515; doi:10.1093/bioinformatics/btae135)
Supplement: btae135_Supplementary_Data [file btae135_supplementary_data.pdf]

Supplementary Material

for

iNGNN-DTI: Prediction of Drug - Target Interaction with Interpretable Nested Graph Neural Network and Pretrained Molecule Models

Yan Sun[1,2,3], Yanyi Li[4], Carson K. Leung[2], Pingzhao Hu[1,2,3,4,5,6,7#]

[1]Department of Biochemistry, Western University, London, Ontario, Canada
[2]Department of Computer Science, University of Manitoba, Winnipeg, Manitoba, Canada
[3]Department of Computer Science, Western University, London, Ontario, Canada
[4]Division of Biostatistics, University of Toronto, Toronto, Ontario, Canada
[5]Department of Oncology, Western University, London, Ontario, Canada
[6]Department of Epidemiology and Biostatistics, Western University, London, Ontario, Canada
[7]The Children's Health Research Institute, Lawson Health Research Institute, London, Ontario, Canada

1. Comparison of performance between the membrane and non-membrane proteins across three datasets

The evaluation results presented by Hegedűs et al. (2022) indicate that AlphaFold2 exhibits comparable performance for membrane proteins as it does for soluble proteins. In our evaluation of membrane proteins within our datasets, we count the predicted Local Distance Difference Test (pLDDT) score. A pLDDT score of 90 or higher is generally considered to indicate very high quality, while scores in the range 70 to 90 are considered confident in the structure predictions. Scores below 70 are indicative of low confidence (Jumper et al., 2021). Specifically, the DAVIS dataset comprises 87 membrane proteins and 355 non-membrane proteins. The BIOSNAP dataset includes 848 membrane proteins and 1267 non-membrane proteins, while the KIBA dataset contains 52 membrane proteins and 176 non-membrane proteins. For each protein, we compute the mean pLDDT score and classify the proteins based on whether their scores are greater than 70 or smaller than 70, as depicted in **Supplementary Figure 1**. In the DAVIS dataset, 97.7% of membrane proteins received scores larger than 70, while 70.4% of non-membrane proteins scored higher than 70. For the BIOSNAP dataset, the percentages are 91.6% and 91.6%, respectively. In the KIBA dataset, the percentages are 92.3% and 83.0%, respectively. Using an Fisher's exact test, we do not observe the significant difference between the membrane and non-membrane proteins in either high confidence or low confidence group for both BIOSNAP data (pvalue = 1.00) and KIBA data (pvalue=0.12), but we do see a significant difference for DAVIS data (pvalue<0.01).

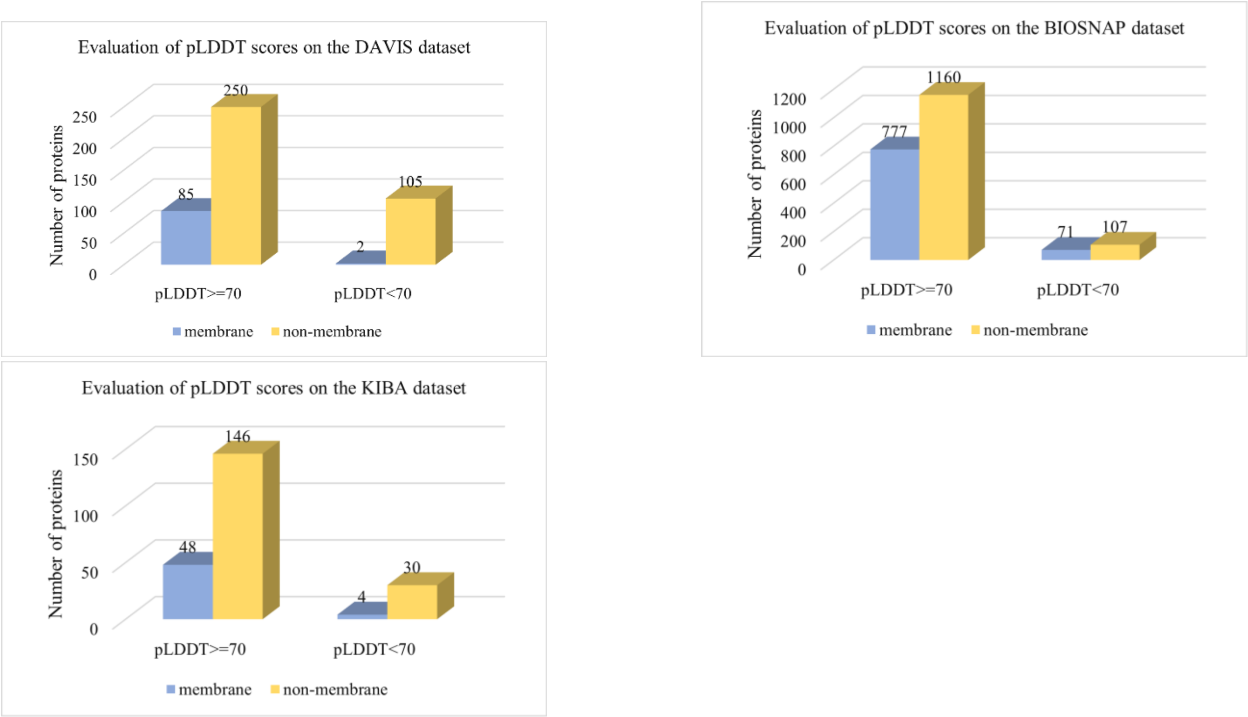

Supplementary Figure 1. Comparison of pLDDT scores between membrane and non-membrane proteins across three datasets.

Furthermore, we conducted a comparison of the structures of two membrane proteins (MEK6 and EPHA2) predicted using the AlphaFold with their true structures using PyMol alignment. The results are presented in **Supplementary Figure 2**. The root mean square distance (RMSD) scores for the two examples are 0.71Å and 0.667Å, respectively. RMSD serves as a metric for assessing structural similarity by calculating the distance between equivalent atoms in two structures. Generally, an RMSD smaller than 2Å is considered indicative of good structural alignment (Carugo & Pongor, 2001; Castro-Alvarez et al., 2017). The results suggest that the AlphaFold can accurately predict the structure of the membrane proteins.

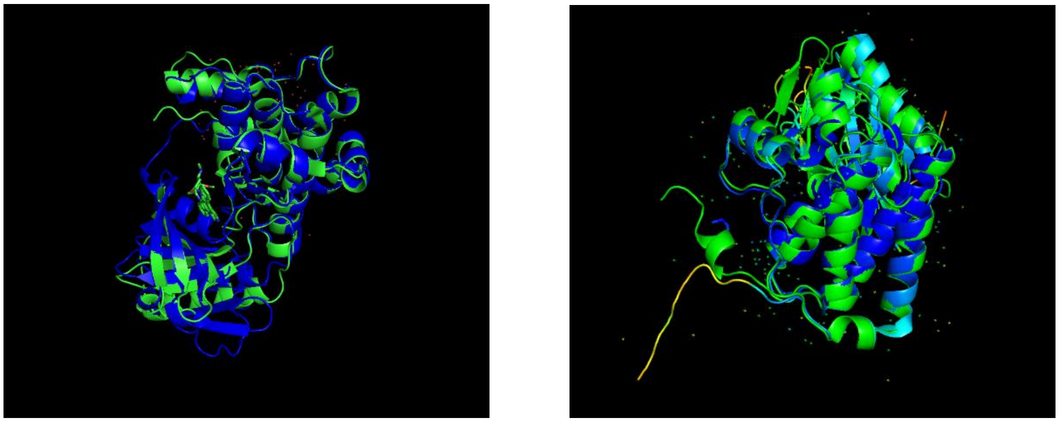

(a) PDB ID: 3FME (RMSD = 0.71)   (b) PDB ID: 5I9X (RMSD = 0.667)

Supplementary Figure 2. Visualization of the comparison between AlphaFold predicted structures and the structures from the Protein Data Bank (PDB). The blue structure represents the AlphaFold predicted structure, whereas the green structure corresponds to the structure from the PDB.

2. Performance comparison of two different contact map generation methods

With AlphaFold2 showcasing impressive accuracy in predicting protein structures, it is anticipated that the resulting contact map would exhibit a high level of quality. We experiment with the SPOT-Contact-LM to predict the contact map and replace the contact map generated by AlphaFold2 using the DAVIS data set. However, as shown in **Supplementary Figure 3**, this configuration results in an AUROC (area under the ROC curve) of 0.924±0.0064 (mean ± standard deviation) and an AUPRC (area under the precision-recall curve) of 0.458±0.0127, which is slightly worse than that using the contact map directly generated by AlphaFold2 (0.931 ± 0.0027 for AUROC and 0.473 ± 0.0167 for AUPRC).

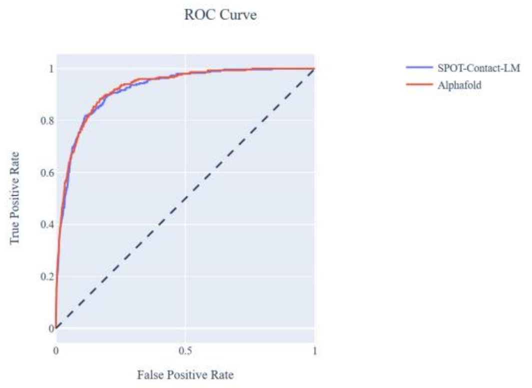

(a) ROC Curve

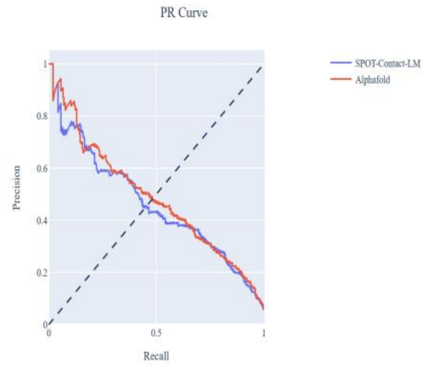

(b) PR Curve

Supplementary Figure 3. ROC curve and PR curve for the two different contact map generation methods.

3. Performance comparison of the DAVIS dataset with varied cut-offs for binarization of the contact map.

Duarte et al. (2010) explored the impact of the cut-off on the contact map for protein structure reconstruction. The findings indicate that beyond 8Å, there is no significant improvement in the accuracy of the reconstructed protein structure. This suggests that a larger threshold may not contribute additional information to the protein structure. Additionally, we conduct experiments with two additional cut-offs, specifically 6Å and 10Å, on the DAVIS dataset. As demonstrated in **Supplementary Table 1**, the performances based on both the 6Å and 10Å cut-offs slightly decrease comparing with those based on the 8Å cut-off.

Supplementary Table 1. Performance on the DAVIS dataset with varied cut-offs for binarization of the contact map.

Threshold	6Å	8Å	10Å
AUROC	0.928 ± 0.0045	0.931 ± 0.0027	0.923 ± 0.0063
AUPRC	0.472 ± 0.0142	0.473 ± 0.0167	0.447 ± 0.0244

References

Carugo, O., & Pongor, S. (2001). A normalized root-mean-square distance for comparing protein three-dimensional structures. Protein science 10(7), 1470–1473. https://doi.org/10.1110/ps.690101

Castro-Alvarez, A., Costa, A. M., & Vilarrasa, J. (2017). The Performance of Several Docking Programs at Reproducing Protein-Macrolide-Like Crystal Structures. Molecules (Basel, Switzerland) 22(1), 136. https://doi.org/10.3390/molecules22010136

Duarte, J.M., Sathyapriya, R., Stehr, H. et al. Optimal contact definition for reconstruction of Contact Maps. BMC Bioinformatics 11, 283 (2010). https://doi.org/10.1186/1471-2105-11-283

Hegedűs, T., Geisler, M., Lukács, G. L., & Farkas, B. (2022). Ins and outs of AlphaFold2 transmembrane protein structure predictions. Cellular and molecular life sciences. CMLS 79(1), 73. https://doi.org/10.1007/s00018-021-04112-1.

Jumper, J., Evans, R., Pritzel, A. et al. (2021). Highly accurate protein structure prediction with AlphaFold. Nature 596, 583–589 (2021). https://doi.org/10.1038/s41586-021-03819-2

Singh, J., Litfin, T., Singh, J., Paliwal, K., Zhou, Y. (2022). SPOT-Contact-LM: improving single-sequence-based prediction of protein contact map using a transformer language model. Bioinformatics 38, 1888–1894.
